# The mediating role of cognitive schemas in the relationship between parenting styles and suicidal ideation in adolescents: a structural equation modeling approach

**DOI:** 10.1186/s13104-025-07514-7

**Published:** 2025-11-05

**Authors:** Fahimeh Alsadat Hosseini, Azita Jaberi, Sara Shaygan, Saghar Salari, Maryam Shaygan

**Affiliations:** 1 Community Based Psychiatric Care Research Center, School of Nursing and Midwifery, Shiraz University of Medical Sciences, Shiraz, Iran; 2 General Department of Education of Fars Province, Shiraz, Iran; 3 Nursing instructor, School of Nursing and Midwifery, Shiraz University of Medical Sciences, Shiraz, Iran

**Keywords:** Adolescents, Maladaptive schema, Parenting style, Suicidal ideation

## Abstract

**Background and aim:**

Suicide is the second leading cause of death among adolescents worldwide, highlighting the urgent need for effective prevention. Parenting styles influence early maladaptive cognitive schemas, which in turn may affect suicidal ideation during adolescence. This study investigated the mediating role of cognitive schemas in the relationship between parenting styles and suicidal ideation among students.

**Methods:**

This cross-sectional study employed structural equation modeling (SEM) on data collected from 593 high school and college students aged 12–21 in Shiraz, Iran, during 2020–2021. Participants were recruited through stratified multi-stage random sampling. Sample size was determined based on SEM guidelines and power analysis. Data were gathered using the Suicidal Ideation Questionnaire, Young’s Schema Questionnaire, and Parental Authority Questionnaire. Analyses were conducted using SPSS 22 and MPLUS. SEM examined direct, indirect, and total effects of parenting styles on suicidal ideation via cognitive schemas.

**Results:**

Five models indicated that authoritarian parenting increased suicidal ideation through all early maladaptive schemas: disconnection/rejection (β = 0.184), impaired autonomy/performance (β = 0.115), other-directedness (β = 0.078), over-vigilance/inhibition (β = 0.052), and impaired limits (β = 0.107). Permissive parenting increased suicidal ideation through disconnection/rejection (β = 0.072), impaired autonomy/performance (β = 0.033), over-vigilance/inhibition (β = 0.030), and impaired limits (β = 0.055) schemas. In contrast, authoritative parenting showed a protective effect by reducing disconnection/rejection schema, thus lowering suicidal ideation (β = -0.055).

**Conclusions:**

Authoritarian and permissive parenting styles increase suicidal ideation through maladaptive schemas, while authoritative parenting acts as a protective factor. These findings provide important insights for designing targeted interventions to reduce suicidal ideation among adolescents.

## Introduction

Adolescence, spanning from puberty to the mid-20s, is a crucial period of psychological and emotional development [1]. This stage is particularly vulnerable to psychiatric disorders, which can impair psychosocial functioning and increase the risk of suicide, a leading cause of death among young individuals [2–4]. Between 2000 and 2021, suicide rates among adolescents increased by 52.2% [5]. In Iran, a systematic review found that individuals aged 15–24 years accounted for 50% of attempted suicides [6]. Suicidal ideation, defined as persistent self-harm-related thoughts, is a significant predictor of suicide attempts and completed suicide [7–9].

Parenting styles play a pivotal role in adolescent emotional development [10]. Defined by parental attitudes and behaviors, they are categorized into authoritarian, authoritative, and permissive [11]. Authoritarian parenting enforces strict obedience, authoritative parenting balances warmth with discipline, and permissive parenting involves minimal rules, potentially leading to impulsivity and emotional instability [12]. These styles influence behavioral and emotional difficulties, including suicidal tendencies [13, 14]. Darvishi et al. (2023) found that authoritarian and authoritative parenting styles have direct positive and negative effects, respectively, on suicidal ideation [15]. While prior research has examined direct influences, fewer studies have explored underlying mechanisms [16–18]. Parenting styles may shape suicidal ideation through their effects on self-esteem [15], parental attachment [18], and gratitude [17]. Recent research by Hatami Nejad et al. (2025) highlights how childhood trauma and victimization influence maladaptive coping through insecure attachment, supporting the role of parenting styles in shaping emotional vulnerabilities linked to suicidal ideation [19].

Cognitive factors, particularly early maladaptive schemas (EMSs), are also implicated in suicidal ideation. Young’s schema theory posits that maladaptive cognitive frameworks formed in childhood influence emotional and behavioral functioning [20]. EMSs, defined as pervasive dysfunctional cognitive patterns, are categorized into five domains: disconnection/rejection, impaired autonomy/performance, over-vigilance/inhibition, other-directedness, and impaired limits [21, 22]. These schemas, often rooted in early negative experiences, shape perceptions, behaviors, and psychological well-being [23]. Studies suggest that schemas such as disconnection/rejection strongly correlate with suicidal ideation and high-risk behaviors among adolescents [23, 24].

While the independent effects of parenting styles and EMSs on suicidal ideation have been explored, limited research has examined their interconnection. Parenting styles significantly shape EMS development [25–28]. Authoritative parenting negatively correlates with maladaptive schemas, whereas authoritarian parenting positively correlates with them [28]. Similarly, rejection and over-control predict various maladaptive schemas [25]. Young’s framework suggests that EMSs mediate the link between parenting styles and emotional distress [26, 29]. Several studies confirm this mediating role in depression [30], anxiety [31], and chronic pain [28]. However, despite EMSs being linked to various psychological disorders, their mediating role between parenting styles and suicidal ideation remains unexplored.

**Fig. 1:**
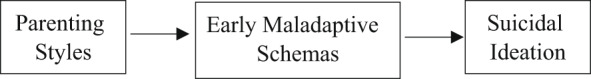
Conceptual model of the study

Given the increasing prevalence of adolescent suicidal ideation [17], understanding how parenting styles influence suicidal thoughts is essential. Developing theoretical models that clarify these pathways can inform targeted interventions [32]. Young’s framework suggests that maladaptive schemas formed through early experiences contribute to psychopathology [33]. Despite studies examining aspects of these relationships independently, a gap persists in understanding how EMSs mediate the link between parenting styles and suicidal ideation. This study aims to address this gap by investigating the mediating role of EMSs in this relationship, hypothesizing that maladaptive schemas serve as key intermediaries through which negative parenting styles elevate suicidal ideation. The conceptual model of the study is illustrated in Fig. 1.

## Methods

### Study design

This study utilized a cross-sectional research design and applied structural equation modeling (SEM) to examine the relationships between parenting styles, cognitive schemas, and suicidal ideation.

### Setting

The research was conducted in Shiraz, the largest city in southern Iran, from August 2020 to March 2021, involving adolescents aged 12 to 21 years.

### Participants

Adolescence, defined as the period between 12 and 21 years [2], is a critical stage of psychological development and heightened vulnerability to emotional difficulties, including suicidal thoughts [34]. Due to the increased risk of suicidal ideation during this developmental period, this study targeted students within this age range [5]. Participants were required to be enrolled in a school or college in Shiraz and willing to complete an online questionnaire. Exclusion criteria included a history of chronic physical illness, diagnosed mental health disorders, or lack of parental contact.

### Sample size

For SEM analysis, a minimum of 20 participants per parameter is recommended [35, 36]. Given the study’s inclusion of at least 20 parameters, a minimum sample size of 400 was required. Based on an estimated 30% prevalence of suicidal ideation among adolescents in Shiraz [37], 750 adolescents were initially recruited through multi-stage sampling. After accounting for dropouts, the final sample included 593 participants, with a mean age of 16.52 ± 3.11 years. To ensure statistical power, a power analysis using G*Power (version 3.1.3) was conducted [38], confirming adequacy for detecting a medium effect size (q = 0.30) with a power of 0.95 (α = 0.05) [39].

### Sampling procedure

The study employed a random, stratified, multi-stage sampling method to select 750 adolescents, including 310 high school students (ages 12 to 18) and 440 college students (ages 18 to 21), based on their population sizes. For school students, a list of schools in four districts was compiled, and four schools (two all-female and two all-male from both first- and second-rank high schools) were randomly selected from each district. Within each school, all students were assigned a code, which was used for random selection via software. For college students, 17 faculties in Shiraz were chosen, and students were randomly selected from each faculty using the same method. After selection, contact information was obtained through school or college officials, and consent forms were sent to parents (for school students) and students (for both groups). Upon consent, the adolescents completed the study questionnaires.

### Ethical considerations and data collection

The study was approved by the ethics committee of Shiraz University of Medical Sciences (IR.SUMS.NUMIMG.REC.1400.030). Participants were provided with a letter detailing the study’s aims, confidentiality, anonymity, and voluntary participation. Adolescents (and their parents, if under 18) signed consent forms before completing online questionnaires. To reduce dropout rates, reminders were sent twice to those who had not completed the questionnaires. All data was collected anonymously and encrypted to ensure confidentiality.

### Instruments

#### Parental authority questionnaire

The 30-item Parental Authority Questionnaire (PAQ) evaluates authoritarian, authoritative, and permissive parenting styles using a 5-point Likert scale (1 = strongly disagree to 5 = strongly agree) [40]. The dominant style is determined by the highest score. The questionnaire demonstrates strong reliability, discriminant validity [40], and confirmed construct validity through factor analysis [41]. Its Persian version has been psychometrically validated [42]. In this study, Cronbach’s alpha was 0.89, indicating high internal consistency.

#### Young schema questionnaire

The 75-item Young Schema Questionnaire (1994) assesses 15 maladaptive schemas across five domains using a 6-point scale (1 = completely false to 6 = completely true) [24]. Its validity and reliability have been established in multiple studies [43–46]. Khosravani et al. confirmed the strong reliability (Cronbach’s alpha = 0.75–0.91) and validity of the Persian version [47]. In this study, Cronbach’s alpha was 0.96, indicating excellent internal consistency.

#### Beck suicidal ideation questionnaire

This 19-item measure assesses suicidal ideation, attitudes, and behaviors. The first five items serve as screening questions; a response indicating suicidal tendencies leads to completion of the remaining 14 questions, each rated from 0 (none) to 2 (high). Each item is scored from 0 (none) to 2 (a lot), yielding a total score range of 0 to 38 [48, 49]. The Beck Suicidal Ideation Questionnaire is a reliable and valid screening tool [50–52], with the Persian version demonstrating strong psychometric properties, including internal consistency (Cronbach’s alpha >0.80) [53] and convergent validity (*r* = 0.76 with the General Health Questionnaire) [54]. In this study, internal consistency was excellent (Cronbach’s alpha = 0.91).

#### Statistical analysis

Descriptive statistics (means, standard deviations, and frequencies) were used to summarize demographic characteristics. Data normality was assessed using skewness (− 2 to + 2) and kurtosis (− 7 to + 7) [55, 56]. Multicollinearity was evaluated using Pearson’s correlations and variance inflation factors (VIF < 2.023).

SEM was conducted using SPSS (version 22) and Mplus (version 8.3) to examine direct, indirect, and total effects of parenting styles on suicidal ideation. Missing data (7.5%) were handled using multiple imputation [57]. Five SEM models were tested, each assessing mediation effects of specific cognitive schemas. Model fit was evaluated using standard indices: CFI and TLI (>0.90), RMSEA (< 0.06), SRMR (< 0.08), AIC, and BIC [58]. The bootstrap method (5,000 samples) was applied to estimate 95% bias-corrected confidence intervals for mediation effects.

## Results

### Participant characteristics

Of the 750 adolescents randomly selected, 157 were excluded—97 due to non-return of consent forms or questionnaires and 60 for ineligibility. The final sample comprised 593 adolescents (mean age = 16.52 ± 3.11 years), with 79.6% being female. Most parents had a high school education or lower (mothers: 61%, fathers: 55.1%). While 68% of mothers were unemployed, 83% of fathers were employed (Table 1).

**Table 1:** Sample characteristics (*n* = 593)

Variables	Value, *n* (%)
Gender	
Female	355 (59.87)
Male	238 (40.13)
Mother’s education	
High school or less	344 (60.99)
University	220 (39.01)
Father’s education	
High school or less	311 (55.14)
Diploma	2 (0.36)
University	251 (44.50)
Mother’s job	
Worker/employer	153 (26.94)
Retired	29 (5.10)
Unemployed	386 (67.96)
Father’s job	
Worker/employer	470 (83.04)
Retired	80 (14.13)
Unemployed	16 (2.83)

### Preliminary data analysis

Normality, outliers, and multicollinearity assessments showed no major concerns. Pearson correlation coefficients remained below 0.7 (Table 2), and VIF values ranged from 1 to 2.023, indicating no multicollinearity issues. Age was significantly associated with permissive and authoritarian parenting styles (*P* < 0.01) and four cognitive schemas (*P* < 0.05), warranting its inclusion as a covariate in mediation analyses. No other socio-demographic variables showed significant links to suicidal ideation, parenting styles, or cognitive schemas (Table 2).

**Table 2:** Bivariate correlations between study variables

Variables	(1)	(2)	(3)	(4)	(5)	(6)	(7)	(8)	(9)	(10)	(11)	(12)	(13)	(14)	(15)
(1) Age	1	0.079	− 0.006	0.082*	− 0.068	0.138 **	0.098*	0.028	0.087*	0.095*	0.100*	− 0.012	0.058	0.086*	0.008
(2) Gender		1	0.063	− 0.024	− 0.051	0.002	0.045	− 0.013	0.060	0.049	0.064	− 0.029	0.027	− 0.027	− 0.060
(3) Mother’s education			1	0.364**	− 0.364**	− 0.039	0.038	0.062	0.043	0.028	0.018	0.022	0.016	0.067	0.002
(4) Father’s education				1	− 0.143	− 0.068	− 0.041	0.014**	0.004	− 0.017	− 0.025	− 0.007	− 0.047	0.045	0.021
(5) Mother’s job					1	0.048	− 0.074	− 0.062	− 0.055	− 0.028	− 0.039	− 0.049	0.072	− 0.062	− 0.03
(6) Father’s job						1	0.037	0.021	− 0.010	0.05	0.063	− 0.013	− 0.009	0.005	0.009
(7) Disconnection and rejection							1	0.705**	0.599**	0.608**	0.579**	0.484**	0.306**	0.516**	0.053
(8) Impaired autonomy								1	0.566**	0.678**	0.486**	0.394**	0.262**	0.437**	0.085*
(9) Impaired limits									1	0.558**	0.621**	0.342**	0.452**	0.524**	0.287**
(10) Other-directedness										1	0.580**	0.301**	0.334**	0.438**	0.232**
(11) Over-vigilance and inhibition											1	0.257**	0.387**	0.399**	0.290**
(12) Suicidal ideation												1	0.144**	0.352**	− 0.068
(13) Permissive													1	0.457**	0.603**
(14) Authoritarian														1	0.178**
(15) Authoritative															1

**The correlation is significant at the 0.01 level. *The correlation is significant at the 0.05 level

### Path analysis results

#### Path model 1: disconnection/rejection schema as a mediator

**Fig. 2:**
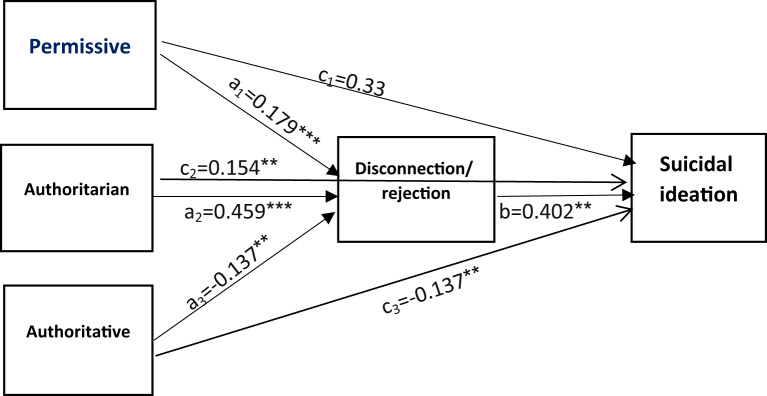
The mediation role of disconnection/ rejection cognitive schema in the relationship between three parenting styles and suicidal ideation. Age was controlled in the model. ***P≤0.001; **P≤0.01; *P<0.05

This model demonstrated a good fit (Table 3). Significant direct effects were found for permissive (β = 0.179, 95% CI 0.085–0.274), authoritarian (β = 0.459, 95% CI 0.388–0.529), and authoritative (β = -0.137, 95% CI -0.222 to -0.051) parenting styles on disconnection/rejection schema. The direct effect of disconnection/rejection on suicidal ideation was significant (β = 0.402, 95% CI 0.325–0.479) (Table 4). Authoritarian (β = 0.154, 95% CI 0.068–0.240) and authoritative (β = -0.137, 95% CI -0.224 to -0.049) parenting styles had significant direct effects on suicidal ideation, while permissive parenting did not (β = 0.033, 95% CI -0.065 to 0.131). The indirect effects of permissive (β = 0.072, 95% CI 0.031–0.113), authoritarian (β = 0.184, 95% CI 0.138–0.230), and authoritative (β = -0.055, 95% CI -0.091 to -0.019) parenting styles on suicidal ideation via disconnection/rejection were significant (Table 4; Fig. 2).

**Table 3:** Fit statistics of estimated models

Statistic	Model 1	Model 2	Model 3	Model 4	Model 5
χ2	10.631	6.549	9.923	10.490	8.745
df	5	5	5	5	5
CFI	0.985	0.994	0.981	0.979	0.990
TLI	0.964	0.986	0.955	0.949	0.975
SRMR	0.034	0.026	0.033	0.034	0.032
RMSEA	0.044	0.023	0.041	0.043	0.036
AIC	12167.628	11920.254	11300.405	11407.182	11229.461
BIC	12224.636	11977.261	11357.413	11464.190	11286.469

*N* = 593 participants. *χ*2: Chi-square test statistic, *df*: Degree of freedom, *CFI*: Comparative fit index, *TLI*: Tucker-Lewis index, *SRMR*: Standardized root mean square residual, *RMSEA*: Root-mean-square error of approximation, *AIC*: Akaike’s information criterion, *BIC*: Bayesian information criterion

**Table 4:** Path coefficients of direct and indirect effects among variables of model 1 (*N* = 593)

Effect	path	Independent variable	Mediating variable	Dependent variable	B	β	SE	95% CI
Direct effect	a_1_	**Permissive**		**Disconnection/ rejection**	0.673***	0.179	0.048	[0.085, 0.274]
	a_2_	**Authoritarian**		**Disconnection/ rejection**	1.255***	0.459	0.036	[0.388, 0.529]
	a_3_	**Authoritative**		**Disconnection/ rejection**	-0.407**	-0.137	0.044	[-0.222, -0.051]
	b	**Disconnection/ rejection**		**Suicidal ideation**	0.116***	0.402	0.039	[0.325, 0.479]
	c_1_	**Permissive**		**Suicidal ideation**	0.035	0.033	0.050	[-0.065, 0.131]
	c_2_	**Authoritarian**		**Suicidal ideation**	0.121***	0.154	0.044	[0.068, 0.240]
	c_3_	**Authoritative**		**Suicidal ideation**	-0.117**	-0.137	0.045	[-0.224, -0.049]
Indirect effect	a_1_b	**Permissive**	**Disconnection/ rejection**	**Suicidal ideation**	0.078***	0.072	0.021	[0.031, 0.113]
	a_2_b	**Authoritarian**			0.145***	0.184	0.024	[0.138, 0.230]
	a_3_b	**Authoritative**			-0.047**	-0.055	0.018	[-0.091, -0.019]

B = Unstandardized coefficients; β = Standardized coefficients; CI 95% Confidence interval; SE = Standard error; ***P ≤ 0.001; **P ≤ 0.01; *P < 0.05. Zero not include in 95% credible interval. Age was controlled in the model

#### Path model 2: impaired autonomy/performance schema as a mediator

**Fig. 3:**
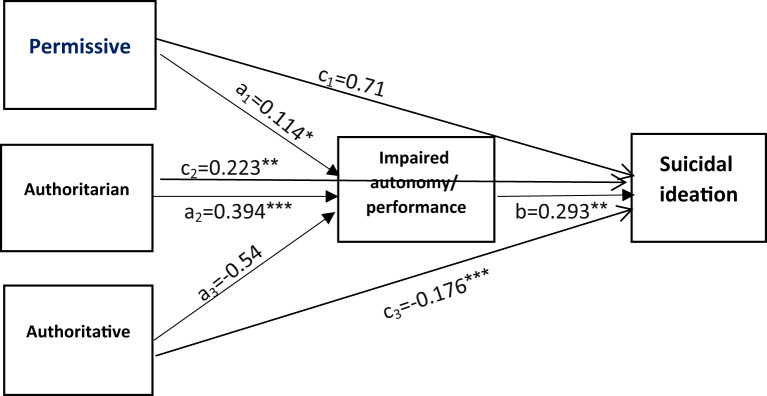
The mediating role of impaired autonomy/ performance cognitive schema in the relationship between three parenting styles and suicidal ideation. Age was controlled in the model. ***P≤0.001; **P≤0.01; *P<0.05

This model also demonstrated a good fit (Table 3). Permissive (β = 0.114, 95% CI 0.013–0.214) and authoritarian (β = 0.394, 95% CI 0.318–0.471) parenting styles significantly predicted impaired autonomy/performance, whereas authoritative parenting did not (β = -0.054, 95% CI -0.145 to 0.038). Impaired autonomy/performance was significantly associated with suicidal ideation (β = 0.293, 95% CI 0.216–0.370) (Table 5). The indirect effects of permissive (β = 0.033, 95% CI 0.003–0.064) and authoritarian (β = 0.115, 95% CI 0.077–0.154) parenting styles on suicidal ideation via impaired autonomy/performance were significant, whereas the indirect effect of authoritative parenting was not (β = -0.016, 95% CI -0.043 to 0.011) (Table 5; Fig. 3).

**Table 5:** Path coefficients of direct and indirect effects among variables of path model 2 (*N* = 593)

Effect	path	Independent variable	Mediating variable	Dependent variable	B	β	SE	95% CI
Direct effect	a_1_	**Permissive**		**Impaired autonomy/ performance**	0.317*	0.114	0.051	[0.013, 0.214]
	a_2_	**Authoritarian**		**Impaired autonomy/ performance**	0.802***	0.394	0.039	[0.318, 0.471]
	a_3_	**Authoritative**		**Impaired autonomy/ performance**	-0.119	-0.054	0.047	[-0.145, 0.038]
	b	**Impaired autonomy/ performance**		**Suicidal ideation**	0.114***	0.293	0.039	[0.216,0.370]
	c_1_	**Permissive**		**Suicidal ideation**	0.077	0.071	0.051	[-0.029, 0.171]
	c_2_	**Authoritarian**		**Suicidal ideation**	0.176 ***	0.223	0.044	[0.137, 0.308]
	c_3_	**Authoritative**		**Suicidal ideation**	-0.151***	-0.176	0.046	[-0.265,-0.086]
Indirect effect	a_1_b	**Permissive**	**Impaired autonomy/ performance**	**Suicidal ideation**	0.036*	0.033	0.016	[0.003,0.064]
	a_2_b	**Authoritarian**			0.091***	0.115	0.020	[0.077,0.154]
	a_3_b	**Authoritative**			-0.013	-0.016	0.014	[-0.043,0.011]

B = Unstandardized coefficients; β = Standardized coefficients; CI 95% Confidence interval; SE = Standard error; ***P ≤ 0.001; **P ≤ 0.01; *P < 0.05. Zero not include in 95% credible interval. Age was controlled in the model

#### Path model 3: other-directedness schema as a mediator

**Fig. 4:**
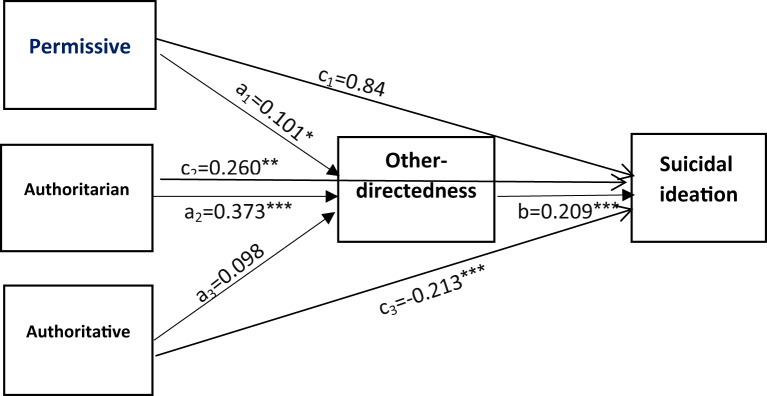
The mediating role of other-directedness cognitive schema in the relationship between three parenting styles and suicidal ideation. Age was controlled in the model. ***P≤0.001; **P≤0.01; *P<0.05

This model exhibited a good fit (Table 3). Significant direct effects were observed for permissive (β = 0.101, 95% CI 0.001–0.200) and authoritarian (β = 0.373, 95% CI 0.297–0.450) parenting styles on other-directedness schema, whereas authoritative parenting was not significant (β = 0.098, 95% CI -0.008 to 0.204). Other-directedness was significantly associated with suicidal ideation (β = 0.209, 95% CI 0.127–0.290) (Table 6). The indirect effect of authoritarian parenting (β = 0.078, 95% CI 0.043–0.112) on suicidal ideation via other-directedness was significant, while the indirect effects of permissive (β = 0.021, 95% CI -0.001 to 0.043) and authoritative (β = 0.021, 95% CI -0.047 to 0.090) parenting styles were not (Table 6; Fig. 4).

**Table 6:** Path coefficients of direct and indirect effects among variables of path model 3 (*N* = 593)

Effect	path	Independent variable	Mediating variable	Dependent variable	B	β	SE	95% CI
Direct effect	a_1_	**Permissive**		**Other-directedness**	0.165*	0.101	0.051	[0.001,0.200]
	a_2_	**Authoritarian**		**Other-directedness**	0.448***	0.373	0.039	[0.297, 0.450]
	a_3_	**Authoritative**		**Other-directedness**	0.148	0.098	0.054	[-0.008, 0.204]
	b	**Other-directedness**		**Suicidal ideation**	0.137***	0.209	0.041	[ 0.127,0.290]
	c_1_	**Permissive**		**Suicidal ideation**	0.091	0.084	0.052	[-0.018,0.186]
	c_2_	**Authoritarian**		**Suicidal ideation**	0.205***	0.260	0.044	[0.174, 0.347]
	c_3_	**Authoritative**		**Suicidal ideation**	-0.183***	-0.213	0.047	[-0.305,-0.122]
Indirect effect	a_1_b	**Permissive**	**Other-directedness**	**Suicidal ideation**	0.023	0.021	0.011	[-0.001,0.043]
	a_2_b	**Authoritarian**			0.061***	0.078	0.018	[0.043,0.112]
	a_3_b	**Authoritative**			0.032	0.021	0.035	[ -0.047,0.090]

B = Unstandardized coefficients; β = Standardized coefficients; CI 95% Confidence interval; SE = Standard error; ***P ≤ 0.001; **P ≤ 0.01; *P < 0.05. Zero not include in 95% credible interval. Age was controlled in the model

#### Path model 4: over-vigilance/inhibition schema as a mediator

**Fig. 5:**
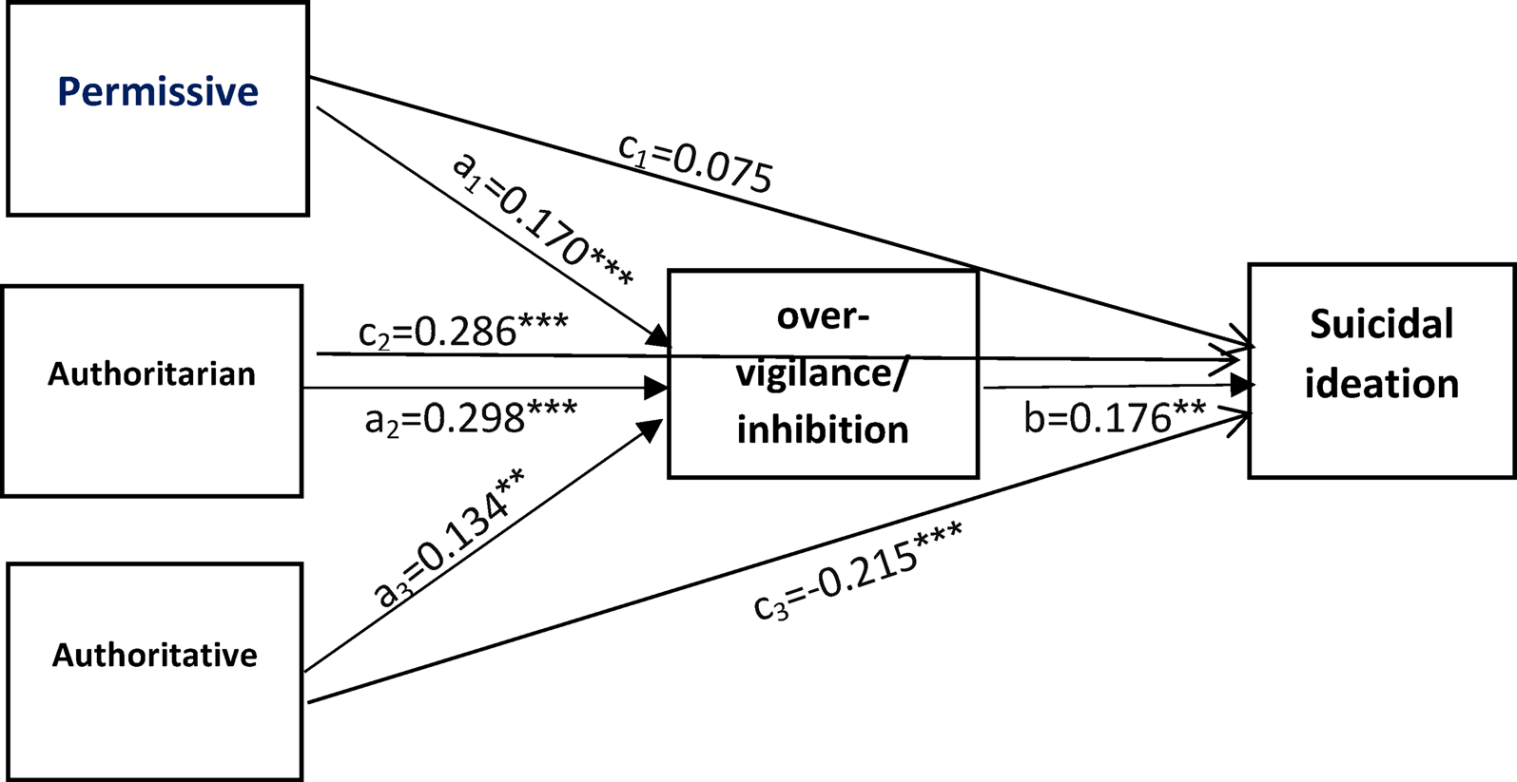
The mediating role of over-vigilance/inhibition cognitive schema in the relationship between three parenting styles and suicidal ideation. Age was controlled in the model. ***P≤0.001; **P≤0.01; *P<0.05

This model demonstrated a good fit (Table 3). Significant direct effects were found for permissive (β = 0.170, 95% CI 0.072–0.269), authoritarian (β = 0.298, 95% CI 0.220–0.376), and authoritative (β = 0.134, 95% CI 0.045–0.224) parenting styles on over-vigilance/inhibition schema. Over-vigilance/inhibition was significantly associated with suicidal ideation (β = 0.176, 95% CI 0.094–0.258) (Table 7). The indirect effects of permissive (β = 0.030, 95% CI 0.008–0.052) and authoritarian (β = 0.052, 95% CI 0.024–0.081) parenting styles on suicidal ideation via over-vigilance/inhibition were significant, whereas the indirect effect of authoritative parenting was not (β = 0.005, 95% CI -0.041 to 0.051) (Table 7; Fig. 5).

**Table 7:** Path coefficients of direct and indirect effects among variables of path model 4 (*N* = 593)

Effect	path	Independent variable	Mediating variable	Dependent variable	B	β	SE	95% CI
Direct effect	a_1_	**Permissive**		**Over-vigilance/inhibition**	0.305***	0.170	0.050	[0.072,0.269]
	a_2_	**Authoritarian**		**Over-vigilance/inhibition**	0.389***	0.298	0.040	[0.220,0.376]
	a_3_	**Authoritative**		**Over-vigilance/inhibition**	0.191**	0.134	0.046	[0.045, 0.224]
	b	**Over-vigilance/inhibition**		**Suicidal ideation**	0.106***	0.176	0.042	[ 0.094,0.258]
	c_1_	**Permissive**		**Suicidal ideation**	0.081	0.075	0.053	[-0.029, 0.178]
	c_2_	**Authoritarian**		**Suicidal ideation**	0.226***	0.286	0.043	[0.201, 0.370]
	c_3_	**Authoritative**		**Suicidal ideation**	-0.185***	-0.215	0.047	[-0.307,-0.123]
Indirect effect	a_1_b	**Permissive**	**Over-vigilance/inhibition**	**Suicidal ideation**	0.032**	0.030	0.011	[0.008,0.052]
	a_2_b	**Authoritarian**			0.041***	0.052	0.014	[0.024,0.081]
	a_3_b	**Authoritative**			0.025	0.005	0.023	[-0.041,0.051]

B = Unstandardized coefficients; β = Standardized coefficients; CI 95% Confidence interval; SE = Standard error; ***P ≤ 0.001; **P ≤ 0.01; *P < 0.05. Zero not include in 95% credible interval. Age was controlled in the model

#### Path model 5: impaired limits schema as a mediator

**Fig. 6:**
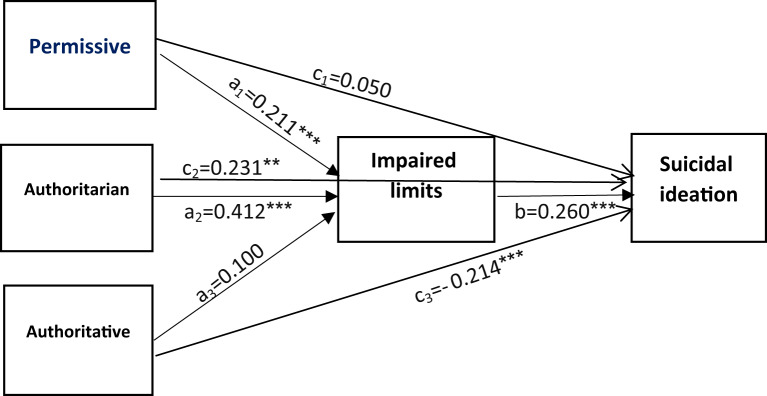
The mediating role of impaired limits cognitive schema in the relationship between three parenting styles and suicidal ideation. Age was controlled in the model. ***P≤0.001; **P≤0.01; *P <0.05

This model also exhibited a good fit (Table 3). Significant direct effects were found for permissive (β = 0.211, 95% CI = 0.120–0.302) and authoritarian (β = 0.412, 95% CI 0.342–0.482) parenting styles on impaired limits schema, whereas authoritative parenting was not (β = 0.100, 95% CI -0.024 to 0.224). Impaired limits schema significantly predicted suicidal ideation (β = 0.260, 95% CI 0.173–0.347) (Table 8). The indirect effects of permissive (β = 0.055, 95% CI 0.025–0.085) and authoritarian (β = 0.107, 95% CI 0.067–0.147) parenting styles were significant, while the indirect effect of authoritative parenting was not (β = 0.023, 95% CI 0.000 to 0.045) (Table 8; Fig. 6).

**Table 8:** Path coefficients of direct and indirect effects among variables of path model 5 (*N* = 593**)**

Effect	path	Independent variable	Mediating variable	Dependent variable	B	β	SE	95% CI
Direct effect	a_1_	**Permissive**		**Impaired limits**	0.356***	0.211	0.046	[0.120,0.302]
	a_2_	**Authoritarian**		**Impaired limits**	0.507***	0.412	0.036	[0.342, 0.482]
	a_3_	**Authoritative**		**Impaired limits**	0.128	0.100	0.063	[-0.024, 0.224]
	b	**Impaired limits**		**Suicidal ideation**	0.167***	0.260	0.044	[ 0.173,0.347]
	c_1_	**Permissive**		**Suicidal ideation**	0.054	0.050	0.053	[-0.053,0.153]
	c_2_	**Authoritarian**		**Suicidal ideation**	0.182***	0.231	0.045	[0.143, 0.320]
	c_3_	**Authoritative**		**Suicidal ideation**	-0.184***	-0.214	0.046	[-0.305,-0.123]
Indirect effect	a_1_b	**Permissive**	**Impaired limits**	**Suicidal ideation**	0.059***	0.055	0.015	[0.025,0.085]
	a_2_b	**Authoritarian**			0.084***	0.107	0.021	[0.067,0.147]
	a_3_b	**Authoritative**			0.019	0.023	0.012	[0.000, 0.045]

B = Unstandardized coefficients; β = Standardized coefficients; CI 95% Confidence interval; SE = Standard error; ***P ≤ 0.001; **P ≤ 0.01; *P < 0.05. Zero not include in 95% credible interval. Age was controlled in the model

In all models, the total effects of permissive (β = 0.105, 95% CI 0.001–0.209), authoritarian (β = 0.338, 95% CI 0.257–0.419), and authoritative (β = -0.191, 95% CI -0.285 to -0.098) parenting styles on suicidal ideation were significant.

## Discussion

Discussion: Given the high prevalence of suicidal ideation among adolescents [10] and its strong link to suicide attempts [11, 12], identifying influential factors is essential for prevention. This study examined the mediating role of maladaptive schemas in the relationship between parenting styles and suicidal ideation.

The Role of Disconnection/Rejection Schema Authoritarian and permissive parenting styles significantly contributed to suicidal ideation through heightened disconnection/rejection schemas, whereas an authoritative style played a protective role [31]. Previous research supports these associations [26]. Young’s schema theory suggests this schema arises from unmet needs for security and nurturance in families marked by detachment or rejection [23]. Authoritarian parents, using punitive and emotionally distant behaviors, may instill a sense of abandonment, increasing vulnerability to suicidal thoughts [59]. The Interpersonal Theory of Suicide [60] and the Integrated Motivational-Volitional Model [61] highlight social connectedness as a protective factor. Authoritarian parenting may weaken family bonds, heightening suicidal risk, while permissive parenting fosters unmet emotional needs, reinforcing maladaptive schemas [62].

The Role of Impaired Autonomy/Performance Schema Authoritarian and permissive parenting styles also influenced suicidal ideation through the impaired autonomy/performance schema [31]. Young’s theory attributes this schema to family environments lacking autonomy support [23]. Adolescents raised under rigid control and minimal emotional support struggle with self-esteem, increasing suicidal risk [60]. Permissively raised adolescents, lacking guidance, face decision-making difficulties. Cognitive theories of depression link incompetence perceptions to sustained depressive cycles and increased suicide risk [63]. Self-determination theory suggests parenting styles fostering autonomy and structure improve adolescent psychological well-being [64].

The Role of Other-Directedness Schema Authoritarian parenting significantly increased suicidal ideation via the other-directedness schema [31, 65]. Young’s theory indicates this schema develops in authoritarian households prioritizing obedience over emotional validation [23]. Adolescents suppress personal needs to gain approval, leading to emotional distress and suicide risk [66].

The Role of Over-Vigilance/Inhibition Schema Authoritarian and permissive parenting styles contributed to suicidal ideation via over-vigilance/inhibition schema [16, 30, 33]. This schema arises in families with inflexible expectations or punitive discipline [23]. Authoritarian parenting fosters perfectionism and emotional suppression, heightening depression and suicidal risk [31]. Emotional inhibition correlates with mental health disorders and suicide risk [67]. Permissive parenting may foster insecure emotional bonds, leading to maladaptive coping [68, 69]. Cognitive styles marked by hypercriticalness and unrelenting standards further contribute to suicidal ideation [70]. However, inconsistencies in permissive parenting findings necessitate further study [31, 65].

The Role of Impaired Limits Schema Both authoritarian and permissive parenting styles increased suicidal ideation via impaired limits schema [26]. Young’s theory attributes this schema to overindulgence or excessive control, leading to self-regulation difficulties [23]. Adolescents from permissive households may struggle with impulse control, increasing emotional instability [71, 72]. Authoritarian parenting suppresses self-expression, reinforcing suicidal ideation [23]. Klonsky and May’s three-step model of suicide [73] highlights interpersonal frustration as a suicide risk factor.

Implications and Limitations This study supports the direct and mediated effects of parenting styles on suicidal ideation within Young’s framework [33]. Findings emphasize adverse parenting’s role in suicide prevention. However, COVID-19’s impact on adolescent mental health should be considered, given increased suicide risk during the pandemic [74–76]. Socioeconomic disparities [9], gender differences [77], and social support [78] also influence suicide risk. The Beck Suicidal Ideation Questionnaire was used due to its psychometric validity in the Iranian population. Future research should examine maternal and paternal parenting differences [79–81] and distinguish between indulgent and neglectful parenting styles.

## Conclusion

This study provides evidence of parenting’s impact on adolescent suicidal ideation, mediated by early maladaptive schemas. Authoritarian parenting heightened suicidal ideation via all schemas, while permissive parenting did so except through the other-directedness schema. In contrast, authoritative parenting emerged as a protective factor, reducing suicidal ideation by mitigating disconnection/rejection schema. These findings inform interventions aimed at reducing adolescent suicide risk. Further research should explore additional dimensions of parenting and suicidality.

## Data Availability

The datasets used and/or analyzed during the current study are available from the corresponding author on reasonable request.

## Abbreviations

SPSS: Statistical Package for Social Sciences
SEM: Structural Equation Modeling
EMSs: Early Maladaptive Schemas
GSHS: Global School-Based Student Health Survey
VIF: Variance Inflation Factor
TLI: Tucker-Lewis Index
CFI: Comparative Fit Index
RMSEA: Root Mean Square Error of Approximation
SRMR: Standardized Root Mean Square Residual
AIC: Akaike Information Criterion
BIC: Bayesian Information Criterion
